# Spatiotemporal Mapping of Colorectal and Gastric Cancer Incidence in Hamadan Province, Western Iran (2010-2019)

**DOI:** 10.34172/jrhs.2025.185

**Published:** 2025-04-01

**Authors:** Erfan Ayubi, Sharareh Niksiar, Zahra Keshtpour Amlashi, Elaheh Talebi-Ghane

**Affiliations:** ^1^Cancer Research Center, Institute of Cancer, Avicenna Health Research Institute, Hamadan University of Medical Sciences, Hamadan, Iran; ^2^Hamadan Cancer Registry, Hamadan University of Medical Sciences, Hamadan, Iran; ^3^Modeling of Noncommunicable Diseases Research Center, Institute of Health Sciences and Technologies, Avicenna Health Research Institute, Clinical Research Development Unit of Fatemieh Hospital, Hamadan University of Medical Sciences, Hamadan, Iran

**Keywords:** Bayesian analysis, Colorectal cancer, Gastric cancer, Iran, Mapping, Spatiotemporal

## Abstract

**Background:** Exploring the pattern of diseases in space and time enhances our understanding of truly needy areas. The present study aimed to explore spatiotemporal mapping of colorectal cancer (CRC) and gastric cancer (GC) incidence using Bayesian models and space-time scan statistics in Hamadan Province from 2010 to 2019.

**Study Design:** An ecological time-series study.

**Methods:** In this study, the data on CRC and GC cases were obtained from Hamadan cancer registry. The crude standardized incidence ratio (SIR) was calculated for each county per year. Hierarchical Bayesian space-time models were fitted to estimate adjusted SIRs. Space time cluster analysis was performed using space-time scan statistic.

**Results:** A total of 1864 CRC cases and 2340 GC cases were included in the analyses. The central counties, including Hamadan (smoothed SIR range: 1.24-1.28) and Tuyserkan (1.01-1.24), exhibited higher than expected number of CRC cases. Northern counties such as Razan (1.19-1.51) and Kabudarahang (1.21-1.42), along with Nahavand in the south (0.98, 1.53), also showed higher than expected number of GC cases. The most likely spatiotemporal cluster of CRC was identified in Hamadan and Tuyserkan occurring between 2015 and 2019 (relative risk [RR]=1.82, *P*<0.001). The most likely spatiotemporal cluster of GC was identified in Nahavand from 2010 to 2011 (RR=1.87, *P*<0.001).

**Conclusion:** Spatiotemporal inequality in the incidence of CRC and GC was identified in Hamadan province over the past decade. The findings may help to reduce cancer disparities and allocate effective resources in the appropriate region and time in the future.

## Background

 Colorectal cancer (CRC) and gastric cancer (GC) are among the most prevalent cancers in both genders in Iran.^1-3^ Iran has experienced transition in urbanization, population aging, and adoption of Western lifestyle.^4-6^ The incidence of CRC and GC following ongoing demographic and risk factors transitions has increased in recent years.^7,8^ CRC and GC are distributed unevenly in Iran over time. Northern and northwestern provinces have higher incidence rates compared to elsewhere.^9-12^ In other words, the rates of CRC and GC in the northern and northwestern provinces of Iran have similar values and tend to cluster together spatially. In a common sense of spatial epidemiology, identification and assessment of spatial and spatiotemporal clusters in a study space can aid in the monitoring and surveillance of diseases.^13^ Kulldorff’s spatial scan statistic is one of the well-known and frequently used methods for the identification and analysis of disease clusters in space and time.^14-16^

 Although spatial inequality in the incidence of CRC and GC has been identified at the provincial level in Iran, the cancer rates in smaller geographical units (e.g., county, district) may be different compared with rate at the provincial level, known as ecological fallacy.^17,18^ Regardless of geographical units, differences in population size can prevent accurate judgment across locations. Through standardization, the burden of outcomes can be compared across locations.^19^ Another problem that can affect the comparison of rates across locations is space-time autocorrelation in which adjacent and neighboring spatial and temporal units tend to have similar values. The Poisson model is commonly used to analyze rate and count data; however, this model is unable to control the problem of sparse data and space-time autocorrelation effectively. In other words, the predicted variability from the Poisson model may not be the same as the observed reality.^20^ In such cases, using Bayesian hierarchical models by integrating spatial and temporal random effect components into the conventional models (e.g., Poisson model) can provide robust standardized rates.^21,22^ Markov Chain Monte Carlo (MCMC) algorithms are common methods for Bayesian inference. Despite their widespread use, MCMC algorithms suffer from problems such as slow convergence, poor chain mixing, and being time-consuming.^23^ To overcome such problems, a method called integrated nested Laplace approximation (INLA) has been introduced.^24^ The INLA is computationally efficient and its accuracy competes with the accuracy of the MCMC.^2^. Given the aforementioned issues, the present study aimed to explore spatiotemporal mapping of CRC and GC incidence using Bayesian models and space-time scan statistics in Hamadan Province from 2010 to 2019.

## Materials and Methods

###  Study area and data sources

 This ecological study utilized population registry data on CRC and GC at the county level in Hamadan province from 2010 to 2019. Hamedan province spans an area of 19493 km^2^ in western Iran, located at coordinates 34.9737° N and 48.5587° E. The province is divided into 9 counties: Hamedan, Malayer, Nahavand, Tuyserkan, Asadabad, Kabudarahang, Razan, Bahar, and Famenin. Hamedan county serves as the provincial capital. Data on the CRC, GC, and also the population size of each county were obtained upon request from the Health Deputy of Hamadan University of Medical Sciences. The cancer registry file included information on age, gender, method of diagnosis (pathology report, death certificate, and clinical diagnosis), ICD code, name of county, and the patient’s residential address. For this study, data related to ICD code 16 for GC and ICD codes 18, 19, and 20 for CRC were used for both genders across each county.

###  Standardized incidence ratio

The number of observed cases of an outcome (e.g., cancer) in each county (Y_i_) follows a Poisson distribution,

*Y_i_~Poisson(E_i_ θ_i_)*

 Where *θ*_i_ is the relative risk (RR) or standardized incidence ratio (SIR) of the outcome in the county i. For each county i, i = 1,…, n, the SIR is obtained using the following equation:

 SIR_i_ = Y_i_/Ei

 Where E_i_ represents the expected cases, which can be calculated as follows:


$$E_{i}=ni\left(\frac{\sum_{i}Yi}{\sum_{i}ni}\right),\;i=1,2,...I$$

Where Y_i_ is the observed number of cases in county i and n_i_ is the population of county i. An SIR greater than 1.00 indicates that the observed number of cancer cases is higher than the expected number.

###  Spatiotemporal clusters 

 Space-time scan statistics were applied to identify the most likely spatio-temporal clusters.^14-16^ The data used for space-time scan statistics include geographic coordinates (latitude and longitude), population at risk, and the observed cases for each county per year. This statistic uses a number of cylinders to detect potential clusters, where the base of the cylinder is circular or elliptical, and its height corresponds to time. The radius and height of the cylinder continuously change as it scans the geographic area and time to identify space-time clusters (e.g., ones with a higher-than-expected number of cases within a geographic region over specified time frames). Cylinders identified through maximum likelihood estimation are considered potential clusters, demonstrating the lowest probability of random occurrence. Assuming that cases within a window follow a Poisson distribution, the log-likelihood ratio (LLR) is defined as follows:


$$LLR=\frac{{\left(\frac{c}{E\left[c\right]}\right)}^{c}{\left(\frac{C-c}{E\left[C\right]-E\left[c\right]}\right)}^{C-c}}{{\left(\frac{C}{E\left[C\right]}\right)}^{C}}$$

Where c and E[c] represent the number of observed and expected cases in cylinder A, respectively. The C and E[C] represent the total number of observed and expected cases in the study area and time period, respectively. The cylinder with maximum likelihood is the primary cluster. The *P* value was obtained using Monte Carlo hypothesis testing. Parameters used for space-time cluster analysis were as follow: probability model: discrete Poisson, scanning window: cylinder with a circular base, type of analysis: retrospective space-time, maximum spatial cluster size: default value or 50% of the population at risk, maximum temporal cluster size: default value or 50% of the study period, maximum Monte Carlo permutations: 999, and significance level: pseudo *P* value < 0.05. The space-time cluster analysis was performed using the SaTScan software version 9.4.2.

###  Spatiotemporal modeling

 It is expected that geographic data units will demonstrate spatial auto-correlation across temporal dimensions.^23^ It is recommended to include spatial and temporal variability in the standard Poisson model to overcome over-dispersion when estimating the SIR.^23^ Therefore, we have:


$$\log\left(\theta_{ij}\right)=\alpha +v_{i}+u_{i}+\gamma_{j}+\emptyset_{j}$$

Where *α* is a constant that represents the overall RR in the study area. The parameter *v*_i_ represents an unstructured spatial random effect that is an unstructured exchangeable component with prior distribution, which is modeled as an independent and identically distributed normal variable with a mean of zero and variance $\sigma_{v}^{2}$ . The parameter *u*_i_ indicates the structured spatial random effect of region i, to account for the spatial dependence between SIRs. The Besag-York-Mollié (BYM) model is the most common model for explaining the structured spatial random effect.^25^ In this model, *u*_i_ is induced by a conditional autoregressive (CAR) model, and SIR in region i tends to shrink toward values of the neighboring areas with a common boundary. The CAR model is as follows:


$$u_{i}|u_{j\neq i}∼N\left(\frac{\sum_{j\in \delta_{i}}u_{j}}{n_{\delta_{i}}},\frac{\sigma_{u}^{2}}{n_{\delta_{i}}}\right)$$

Where *n*_δi_ is the number of areas that share boundaries with area i.

 The parameter *γ*_j_ represents the structured temporal effect that can be modeled using random walk.^23^ The random walk model effectively captures changes in time series data in a gradual and continuous manner. This model assumes that changes at each time point depend solely on the previous state, thereby reducing computational complexity and facilitating analysis.^23^ For example, random walk in time of second order (RW2) is:


$$\gamma_{j}|\gamma_{j-1}\;\gamma_{j-2}∼∼N(2\gamma_{j-1}-\gamma_{j-2},\sigma_{r}^{2})$$

The Φ_j_ is unstructured temporal effect, $\emptyset_{j}∼N\left(0,\sigma_{\emptyset }^{2}\right)$

 It is expected that the areas will have their own specific temporal patterns while also exhibiting spatial dependence on neighboring areas, a space-time interaction. The classical parametric model to take into account is as follows:


$$\log(\theta_{ij})=\alpha +u_{i}+v_{i}+(\beta +\delta_{i})\times t_{j}$$

Where *β* denotes the main linear time trend that represents the global time effect, and *δ*_i_ denotes a space-time interaction. Another suggested model for accounting for space-time interaction is non-parametric models, as follows:


$$\log(\theta_{ij})=\alpha +u_{i}+v_{i}+\gamma_{j}+\emptyset_{j}+\delta_{ij}$$

Where *δ*_ij_ is the interaction term. Four types of interactions have been proposed,^21^ type I (*v*_i_, Φ_j_), type II (*v*_i_*,γ*_j_), type III (*u*_i_,Φ_j_), and type IV (*u*_i_*,γ*_j_). These interaction terms explain the variations in time trends across different areas and can help account for differences in cancer trends among various counties.^21^

 In this study, 6 Bayesian models were used to explore the spatiotemporal mapping of CRC and GC incidence. The following assumptions were considered: independent and identically distributed (i.i.d) Gaussian prior to unstructured spatial (*v*_i_), independent and identically distributed (i.i.d) Gaussian prior for temporal effect (Φ_j_), CAR prior to structured spatial effect (*u*_i_), and second (RW2) order random walk prior to structured temporal effect (*γ*_j_). The distribution of hyper-parameters σ, of spatial and temporal random effects was specified using the default specification of R-INLA. The spatial adjacency matrix was defined using the queen contiguity criterion. The posterior estimate (mean) of SIRs adjusted for effects of space, time, and space-time interaction were obtained using INLA approach^24^ in R 4.1.3 software. The deviance information criterion (DIC), Watanabe-Akaike information criterion (WAIC),^26,27^ the Σlog conditional predictive ordinate (Σlog CPO), probability integral transform (PIT),^28^ and R^2^ value were the criteria to select the best-fitting model. According to the rule of thumb, the best-fitted model is the one with lower values of DIC and WAIC and a higher value of Σlog CPO and R^2^ compared to other models. Additionally, the PIT values should have a more or less uniform distribution, and the uniformity was tested using the Anderson-Darling test.

## Results

**Figure 1:**
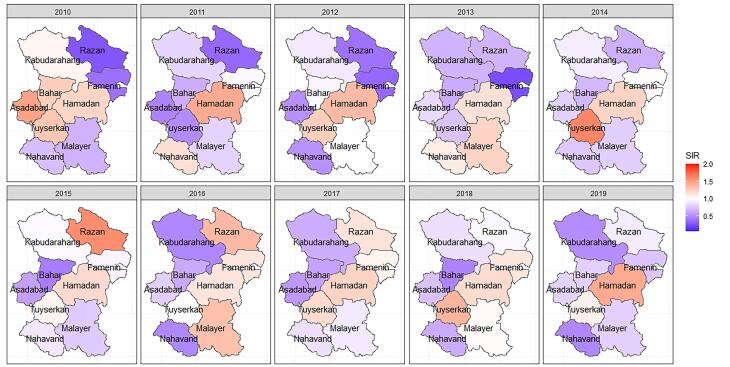
The spatiotemporal mapping of the crude standardized incidence rate of colorectal cancer in Hamadan province during 2010-2019. SIR: Standardized incidence ratio

**Figure 2:**
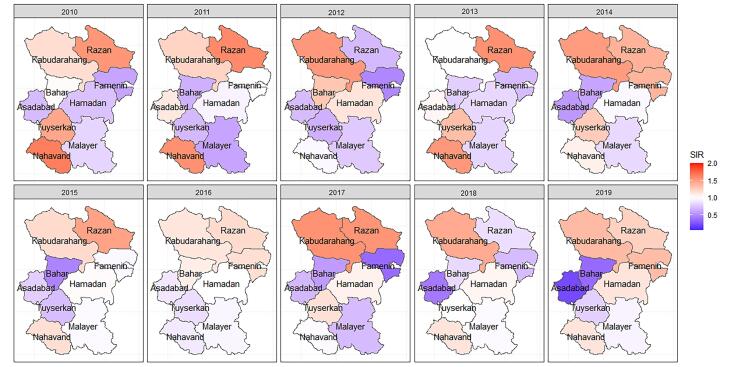
The spatiotemporal mapping of the crude standardized incidence rate of gastric cancer in Hamadan province during 2010-2019, SIR: Standardized incidence ratio

 A total of 1864 cases of CRC and 2340 cases of GC were included in the analysis. The overall incidence rate of CRC was 107.72 per 100 000, and the overall incidence rate of GC was 135.23 per 100 000 people from 2010 to 2019. The crude SIRs of CRC and GC from 2010 to 2019 are illustrated in Figures 1 and 2. Over the decade, a disparity in CRC incidence was observed in Hamadan province, where northern and southern counties reported lower than expected rates. In contrast, central counties such as Hamadan and Tuyserkan exhibited higher than expected number of cases. For instance, in 2019, the crude SIR of CRC in Hamadan county was 1.44 (observed = 150, expected = 104), and it was 1.08 (observed = 19, expected = 17) in Tuyserkan. Additionally, the lowest crude SIR of 0.48 was reported for Nahavand county (observed = 15, expected = 31) in 2019 (Figure 1). There has been a spatial inequality in the incidence of GC in Hamadan province over the past decade, with Razan and Kabudarahang in the north and Nahavand in the south showing higher than the expected number of cases. In contrast, the central counties reported observed cases equal to or below expected levels. In Razan and Kabudarahang, the number of observed cases exceeded expectations in most of the years. For example, the observed number of GC cases in these two counties was about 35% higher than the expected number of cases (SIR = 1.35) in 2019. Recently, there has been a gradual increase in the trend in Nahavand county as well, with the SIR in Nahavand being 1.14 in 2018 and 2019 (Figure 2).

 The space-time scan statistics identified one significant low-risk cluster and one high-risk cluster of CRC during 2010-2019. The low-risk cluster was identified between 2010 and 2014, encompassing Kabudarahang, Famenin, Razan, and Bahar counties. During this period, people in these 4 counties had a 49% lower risk of CRC compared to other areas (RR = 0.51(. In 2015-2019, 394 CRC cases were expected to occur in Hamadan and Tuyserkan counties, but 610 cases were observed (RR = 1.82). For GC, two significant high-risk clusters were identified in 2010-2011. The most likely low-risk cluster was identified in 2015-2019, including Bahar and Asadabad counties in which the risk of GC among residents of these two counties was 37% lower compared to other areas (Table 1).

**Table 1:** The space-time clusters of colorectal and gastric cancers in Hamadan province during 2010-2019

**Cancer**	**County included**	**Time **	**Population**	**Observed**	**Expected**	**O/E**	**RR**	**logLR**	***P *** **value**
Colorectal									
Primary low rate cluster	Kabudarahang, Famenin, Razan, Bahar	2010-2014	410004	118	218.63	0.54	0.51	30.88	0.001
Primary high rate cluster	Tuyserkan, Hamadan	2015-2019	766614	610	393.62	1.55	1.82	67.61	0.001
Gastric									
Primary low rate cluster	Asadabad, Bahar	2015-2019	238236	98	152.33	0.64	0.63	11.77	0.001
Secondary low rate cluster	Malayer	2011-2014	299070	113	160.23	0.71	0.69	8.27	0.030
Primary high rate cluster	Nahavand	2010-2011	193745	96	52.21	1.84	1.87	15.10	0.001
Secondary high rate cluster	Famenin, Kabudarahang, Razan	2010-2011	285358	114	77.19	1.48	1.50	7.94	0.040

RR: relative risk, logLR: log likelihood ratio

**Figure 3:**
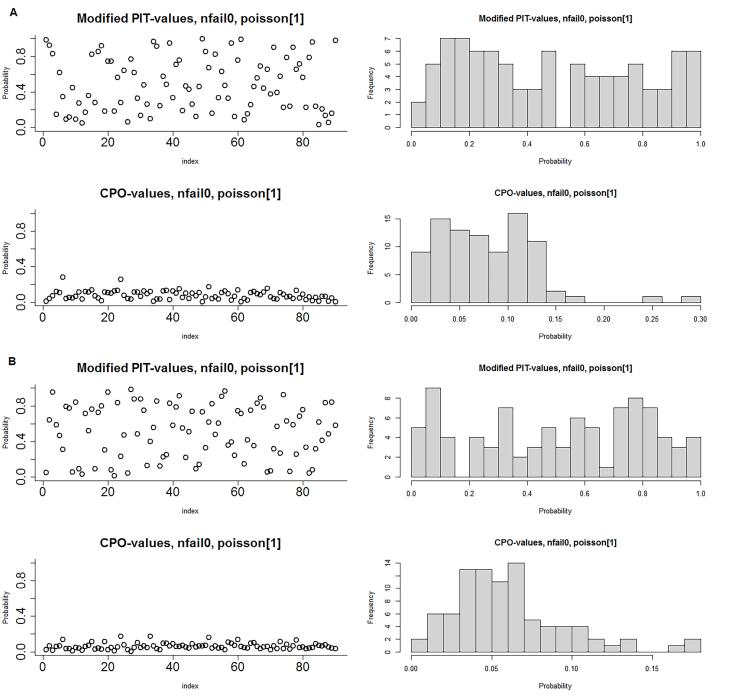
Conditional predictive ordinate (CPO) and probability integral transform (PIT) distribution of type IV space-time interaction structure; A) Colorectal cancer, B) Gastric cancer

Table 2 presents the results of goodness of fit statistics for 6 Bayesian models. It can be inferred that parametric model and non-parametric models including type II and type IV interaction structures fit better on the dataset compared with other models. Nonetheless, type II and type IV interaction structures explain 53% and 46% of the variability in the SIR of CRC and GC, respectively. The CPO and PIT values obtained from type IV interaction structure are depicted in Figure 3. The PIT values exhibit a uniform distribution, and also observations with CPO values different from others are more or less unlikely. The Anderson-Darling test also confirmed the null hypothesis of uniform distribution of type IV interaction structure to model CRC (test statistic = 0.88, *P* = 0.42) and GC (test statistic = 0.68, *P* = 0.57).

**Table 2:** Goodness of fit and performance statistics of six Bayesian Models to assess space-time pattern of colorectal and gastric cancer in Hamadan province during 2010-2019

**Model**	**Colorectal cancer**				**Gastric cancer**			
	**DIC**	**WAIC**	**SumLog(CPO)**	**R**^2^	**DIC**	**WAIC**	**SumLog(CPO)**	**R**^2^
Non-parametric model, no time-space interaction	502.09	503.75	-252.06	0.28	546.27	549.44	-274.91	0.42
Non-parametric model, time-space interaction (Type I)	502.19	503.75	-252.08	0.28	546.21	549.42	-274.92	0.42
Non-parametric model, time-space interaction (Type II)	502.41	505.47	-254.60	0.46	534.57	534.08	-268.09	0.53
Non-parametric model, time-space interaction (Type III)	517.00	521.55	-262.64	0.28	560.34	567.63	-285.44	0.42
Non-parametric model, time-space interaction (Type IV)	502.13	505.10	-254.44	0.46	535.15	534.76	-268.49	0.53
Parametric model	502.87	504.51	-252.55	0.29	534.44	535.93	-268.18	0.46

DIC: deviance information criterion, WAIC: Watanabe-Akaike information criterion, CPO: Conditional Predictive Ordinate, R^2^: r-squared

**Figure 4:**
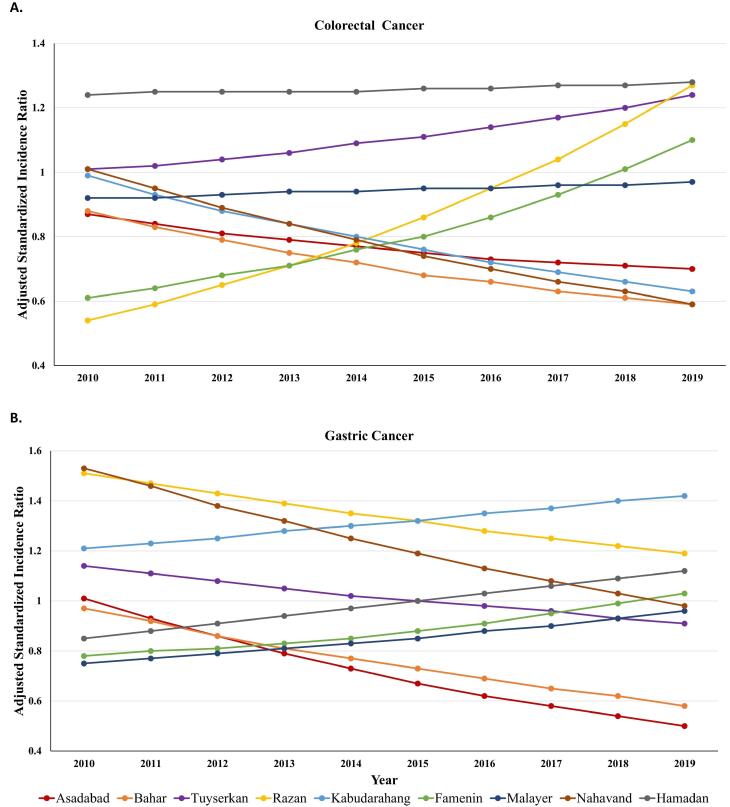
Smoothed standardized incidence ratio of colorectal (A) and gastric (B) cancer trend in Hamadan province during 2010-2019 based on type IV

 The adjusted SIRs stratified by county and year are presented in Figure 4. Over time, the SIR of CRC and GC in the counties underwent changes after adjusting for space, time, and their interaction. The adjusted SIRs of CRC for Hamadan and Tuyserkan were greater than 1.00, fluctuated in all years, and had a gradual increase from 2010 to 2019. This increase was more pronounced in Tuyserkan. These counties had the highest rate of CRC with values of 1.28 and 1.24 in 2019. The SIRs in Razan and Famenin increased so sharply from 2010 until 2019 that adjusted SIRs in both counties exceeded 1 after 2017 and reached 1.27 and 1.10 in 2019, respectively. In other counties, the adjusted SIRs of CRC were below 1.00 during the study years. Malayer had a consistent trend of SIRs during a decade while the other counties had a gradual downward trend until 2019. Bahar and Nahavand had the lowest SIRs in 2019 with a value of 0.59 in both counties (Figure 4A).

 The trends in Figure 4B demonstrated that the adjusted SIRs of GC for Razan, Nahavand, and Kabudarahang were greater than 1 from 2010 to 2019. The trend for the two first counties was downward and it was upward for the third county over time. As observed, the highest SIRs in 2010 were 1.53 and 1.51 for Razan and Nahavand and in 2019 and they were 1.42 and 1.19 for Kabudarahang and Razan. Hamadan and Tuyserkan had completely opposite trends. While the adjusted SIR for Hamadan was less than 1 (0.85) in 2010, it increased continuously from 2010 to 2019 and peaked in 2019 (1.12), the adjusted SIR for Tuyserkan was greater than 1 (1.14) in 2010, declined continuously from 2010 to 2019 and was the lowest in 2019 (0.91). In other counties, the adjusted SIRs of GC were below 1 during a decade. Although Asadabad and Bahar had a gradual downward trend until 2019, Famenin and Malayer had a gradual upward trend of SIRs during the decade. Furthermore, Bahar and Asadabad had the lowest SIRs in 2019 with values of 0.58 and 0.50, respectively (Figure 4B).

## Discussion

 Spatiotemporal models can be used to develop disease distribution maps which are essential to allocate effective resources in the appropriate region and time. The present study enhanced the mapping knowledge of CRC and GC in all counties of Hamadan province over a decade (2010-2019) by employing spatiotemporal Bayesian models and space-time scan statistics. Spatiotemporal inequality in the incidence of CRC and GC was identified, with several significant high-risk and low-risk clusters emerging over time. The central counties of the province exhibited a higher risk of CRC, while the northern and southern counties showed a higher risk of GC.

 The results of the models used in this study demonstrated that the incorporation of temporal and spatial random components along with their interactions into the conventional model (e.g., the Poisson model) improved the performance of mapping. Moreover, to overcome the limited number of cancer cases in some counties which led to sparse data, the Bayesian models were executed using the INLA method without encountering convergence problems. The effectiveness of the INLA method was confirmed in other studies even in cancer datasets with zero-inflation.^29^ Among the developed Bayesian models, the non-parametric models of types II and IV exhibited superior performance compared to the others. Based on model selection criteria (e.g., DIC, WAIC, and CPO), there was only a slight difference between type II and IV models. The analyses highlighted that the cancer incidence rate in a geographic unit tends to shrink toward the incidence rate of neighboring locations and temporal units. The results of the present study, which are similar to previous studies conducted in Iran, indicated that considering spatial and temporal variability for the mapping of cancer is preferable.^29-31^

 Our study results showed that the incidence of CRC is more concentrated in the counties located in the center of the province, while the border counties have lower rates. The findings are consistent with a previous geospatial analysis of CRC in Hamadan province from 2007 to 2014.^32^ In the aforementioned study, the spatial unit was district level and the hot spot clusters were identified in the center of the province, while the significant cold spot clusters were identified in the north and northeast.^32^ The higher risk of CRC in Hamadan county may be attributed to the impact of socioeconomic status (SES). Hamadan county is the capital of Hamadan province, where SES is higher compared to other counties. Higher SES can result in greater health awareness, leading to increased utilization and adherence to CRC screening guidelines.^33,34^

 The high incidence of GC in the northern and southern counties might result from differences in genetic background and exposure to environmental factors such as *Helicobacter pylori* infection, unhealthy diet (e.g., sodium and salted foods), and soil and water pollutants (e.g., heavy metal and chemical composition). The border counties of Hamadan province have a lower SES compared to the counties located in the center of the province. Low socioeconomic conditions, along with poor hygiene and sanitation, predispose individuals to acquire *H. pylori* infection.^35,36^ Previous studies have suggested that the concentration of nitrate in water resources in areas close to the north and south of Hamadan province was higher than in other locations.^37,38^ A study has also suggested a direct relationship between the level of nitrate and lead in water and GC in Hamadan province.^39^

 Several limitations should be acknowledged. First, the results in smaller geographic units, such as census tracts, can differ from those at larger levels, like counties (ecological fallacy). Furthermore, spatial analysis relies on the way boundaries are defined and the methods employed for data aggregation, which highlighting the modifiable areal unit problem. Second, the current study presents a univariate spatiotemporal model; however, some variables such as the SES of counties can affect the spatiotemporal mapping of CRC and GC. It is expected that incorporating exploratory variables into the model design will enhance its predictive power, leading to higher R^2^ values. Third, geocoding was not possible for some cancer cases because the county name was not specified; they were only recorded as cancer cases in Hamadan province. Finally, we defined random effects and hyperparameters using commonly used prior distributions and default choices. However, testing different priors and evaluating their effects on model predictions can help ensure optimal results.

**Highlights**

- Hierarchical Bayesian space-time models can provide new insights into spatiotemporal mapping of cancers.
- The Bayesian models provide evidence for spatial disparity in colorectal cancer (CRC) and gastric cancer (GC) in Hamadan province from 2010 to 2019.
- The central counties exhibited a higher risk of CRC.
- The northern and southern counties showed a higher risk of GC.

## Conclusion

 In this study, Bayesian hierarchical methods and space-time scan statistics were used for spatiotemporal mapping of CRC and GC incidence in Hamadan province from 2010 to 2019. The analyses highlighted the presence of spatiotemporal disparity in the incidence of CRC and GC, where CRC was more concentrated in the counties located in the center of the province and GC was predominantly found in the northern and southern regions. Future studies should focus on the mapping of these two cancers in smaller geographic units, incorporating various prior types for spatial and temporal random components, to enhance insights into the spatiotemporal mapping of CRC and GC.

## Acknowledgments

 The authors would like to thank the Vice-Chancellor of Health of Hamadan University of Medical Sciences for providing the data and the Vice-Chancellor of Research of Hamadan University of Medical Sciences for supporting this research.

## Competing Interests

 The authors declare no competing interests.

## Ethical Approval

 The study was approved by the Research Ethics Committee of the Hamadan University of Medical Sciences (IR.UMSHA.REC.1403.539).

## Funding

 The present study was supported by Hamadan University of Medical Sciences, Hamadan, Iran (Research ID: 140308016665).
